# Chelate facilitated phytoextraction of Pb, Cd, and Zn from a lead–zinc mine contaminated soil by three accumulator plants

**DOI:** 10.1038/s41598-023-48666-5

**Published:** 2023-12-01

**Authors:** Sadegh Hosseinniaee, Mohammad Jafari, Ali Tavili, Salman Zare, Giovanna Cappai

**Affiliations:** 1 Department of Reclamation of Arid and Mountainous Regions, Natural Resources Faculty, University of Tehran, Karaj, Iran; 2 Department of Civil-Environmental Engineering and Architecture, University of Cagliari, Monserrato, Italy

**Keywords:** Ecology, Environmental sciences

## Abstract

This study aims to evaluate the enhancement of phytoextraction of heavy metals (Pb, Cd, and Zn) by species *Marrubium cuneatum*, *Stipa arabica*, and *Verbascum speciosum*, through EDTA amendment. Assisted phytoextraction pot experiments were performed at different EDTA dosages (0, 1, 3, and 5 mmol kg^−1^ soil). The DTPA-extractable metal content increased in the presence of EDTA, followed by their contents in the tissues of all three studied species. Resulting from oxidative stress, the activity of antioxidant enzymes such as glutathione peroxidase (GPX), superoxide dismutase (SOD), and catalase (CAT) increased when the chelating agent was added. EDTA in higher doses partially decreased chlorophyll concentration, and 5 mmol kg^−1^ of that reduced the biomass of the studied species. The bioconcentration factor (BCF) for Cd was notably high in all studied plants and considerably elevated for Zn and Pb with the addition of EDTA in *M. cuneatum* and *S. arabica* (BCF > 1), whilst an accumulation factor greater than one (AF > 1) was found for Cd in all species and for Pb in the case of *S. arabica*. In general, the results demonstrated that EDTA can be an effective amendment for phytoextraction of Cd, Zn, and Pb by *M. cuneatum*, *V. speciosum* and *S. arabica* in contaminated soils.

Recently, a rapid increase in soil metal pollution has resulted from continuing urbanization and industrialization, and as a serious global issue, the dispersion of potentially toxic metals throughout the environment poses a menace to humans and ecosystems^1–4^. There has been a growing concern regarding heavy metal pollution, which is primarily the result of anthropogenic activities such as smelting and mining, effluent discharging, solid waste disposal, and industrial waste treatment^5–7^. A negative effect of potentially toxic metals on soil microbial populations can result in a reduction of biological activity, and it has been shown that a heavy metal's absorption and accumulation inhibit plant growth; these metals also interfere with root and leaf development^8,9^. Heavy metal stress triggers a multitude of complex plant reactions; essentially, these mechanisms involve the concept of irritants as well as antioxidant defenses^10,11^.

Metal-contaminated areas must be remediated due to the environmental and health implications resulting from heavy metal contamination to minimize their effects on the ecosystem. Research on various physicochemical and biological approaches has been conducted over the past few decades^12,13^. Heavy metal-contaminated soils are typically decontaminated using either encapsulation or dig-and-dump methods, but neither of these techniques address the issue of decontamination^14^. Traditional remediation technologies based on chemicals present disadvantages such as high costs, complex technical requirements, and secondary pollution. Also, in most cases, they are only suitable for small contaminated areas that need to be treated quickly and thoroughly^15,16^. Moreover, traditional techniques degrade most of the inherent characteristics of the soil and kill its living organisms^17,18^. Currently, phytoremediation has been confirmed as the most economical and ecologically friendly option. Phytoextraction, a common phytoremediation technique, involves using accumulator species for the removal of pollutants from the soil via the soil-root-to-shoot system and is applicable on large scales^19,20^. Additionally, plant species are ideal candidates for remediation purposes because they are non-disrupting to ecosystems and have been proven to be useful for the in-situ phytoremediation process of a wide range of toxic metals^21^.

As a phytoremediation process, phytoextraction is regarded as the most efficient method for removing soils contaminated with heavy metals and metalloids^12,22^. It typically employs plants with a high growth rate, a developed root system, resistance to heavy metal toxicity, and a large biomass with a high accumulation of metal contents in their aerial and harvestable parts^23^. Different plant species exhibit various phytoextraction potentials for heavy metals, depending on their species, metal availability, and growing environments^10,24^. A plant's phytoextraction potential is mainly determined by its ability to adsorb heavy metals into its harvestable parts, and the DTPA-extractable metal in soil is a critical limitation of phytoextraction^25^. Over the past decades, chelating agents have been extensively studied as a means of increasing the metals' solubility in soil and the ability of plants to adsorb them. Common chelating agents for boosting plants' uptake of heavy metals include nitrilotriacetic acid (NTA), ethylene diamine disuccinic acid (EDDS), ethylenediaminetetraacetic acid (EDTA), and low-molecular-weight organic acids (LMWOAs)^7,26,27^. In the meantime, EDTA is the most effective synthetic chelating agent for enhancing metal availability, sorption, and complexation^28–30^. For the remediation of heavy metal-contaminated soils, EDTA has been widely used with plants such as *Bidens maximowicziana*^31^, *Chrysopogon zizanioides*^1^, and *Brassica juncea*^7^, indicating its enhancement effect on metal absorption.

Selecting suitable plant species is the key factor in successfully implementing phytoremediation, as they must be tolerant of high metal concentrations and compatible with ecological conditions. Native species growing naturally in mining areas are usually metal-adapted, which makes them ideal candidates for phytoremediation. For this study, enhanced phytoextraction was performed on the following plant species: *Stipa arabica* Trin. & Rupr. (Poaceae), *Marrubium cuneatum* Banks & Sol. (Lamiaceae), and *Verbascum speciosum* Schrad. (Scrophulariaceae), which are perennial with high biomass and metal-tolerant. In our previous study to identify native species with high phytoremediation potential, *M. cuneatum* served as a stabilizer and accumulator of Cd and Zn, respectively, and also showed the highest accumulation of Pb in its shoot. *V. speciosum* and *S. arabica*, with a significant accumulation factor (AF) and translocation factor over one (TF > 1), are potentially appropriate plants for Pb and Zn removal through phytoextraction^9^. Hence, the objective of this general research is to assess the effect of the EDTA chelating agent on (1) Cd, Pb, and Zn uptake by *M. cuneatum*, *S. arabica*, and *V. speciosum*, (2) plant growth and physiological characteristics, and (3) determining the optimal dosage for EDTA application. To our knowledge, this is the first study that evaluates the metal uptake by the mentioned species in greenhouse conditions, as well as the effect of EDTA on the growth, physiological response, and phytoextraction capability of these plants.

## Results and discussion

### The effects of various EDTA doses on plant biomass

As shown in Table 1, EDTA affected the growth of the studied plants. The results demonstrated that EDTA treatment in low concentrations shows no significant effect on the root dry weight of the studied plants, and even in *M. cuneatum* and *V. speciosum*, it slightly increased, while during the increase in dose to 5 mmol kg^−1^, the root biomass of *S. arabica*, *V. speciosum*, and *M. cuneatum* decreased by 30, 20, and 22%, respectively, compared with those grown in untreated conditions (Table 1). The dry weight of the shoot was not significantly different between the control and doses of 1 and 3 mmol kg^−1^ EDTA for the studied species. Similar to the roots, at the level of 5 mmol, EDTA inhibited the growth of the shoot of all three species and decreased by 17, 6, and 11%, respectively, in *S. arabica*, *V. speciosum*, and *M. cuneatum* compared to the control; however, the negative effect of EDTA on root growth was more severe than that of the shoot (Table 1). Considering that the roots are exposed to heavy metals first, they are more affected than the shoots^32^. Compared to *V. speciosum*, *S. arabica* and *M. cuneatum* exhibit higher biomass decreases because they contain a higher concentration of heavy metals, which may induce the plants to suffer more stress^33,34^. In this work, the decrease in plant growth due to the application of the above doses of the chelating agent is the result of the high accumulation of potentially toxic metals, which is greater than the plant's capacity to activate the defense system^35,36^. In addition, EDTA could inhibit plant growth by reducing chlorophyll biosynthesis^7^. The negative effects of this synthetic chelator because of its interference with soil fertility were observed by other researchers^37,38^, in contrast, EDTA has been found to improve plant growth^11,39–41^. Regarding this, Saleem et al.^11^ stated that EDTA can increase the chlorophyll concentration of the plant under copper pollution stress, and by increasing the photosynthetic pigments, it causes the effective conversion of light in the photochemical processes of photosynthesis and thus improves the growth and development of the plant. As a consequence, EDTA has variable effects on biomass depending on plant species, soil metal concentration, and dosage of chelating agents.

**Table 1:** Effects of the EDTA application on the dry mass of the studied species (Mean ± SD; n = 3).

Species	*M. cuneatum*		*V. speciosum*		*S. arabica*	
	Root	Shoot	Root	Shoot	Root	Shoot
CK	0.41^a^ ± 0.02	2.56^a^ ± 0.06	0.25^b^ ± 0.002	1.77^ab^ ± 0.06	1.30^a^ ± 0.006	2.08^a^ ± 0.01
E1	0.38^a^ ± 0.003	2.62^a^ ± 0.03	0.27^a^ ± 0.009	1.90^a^ ± 0.09	1.28^a^ ± 0.04	2.13^a^ ± 0.01
E2	0.42^a^ ± 0.01	2.66^a^ ± 0.06	0.24^b^ ± 0.006	1.82^ab^ ± 0.02	1.27^a^ ± 0.02	2.07^a^ ± 0.01
E3	0.32^b^ ± 0.009	2.27^b^ ± 0.07	0.20^c^ ± 0.006	1.66^b^ ± 0.05	0.90^b^ ± 0.02	1.71^b^ ± 0.05

Different lowercase letters in a same column indicate significant differences existed among different plants or soils (P < 0.05, Tukey’s test). CK, E1, E2, and E3 represent soil treated with EDTA of 0, 1, 3, and 5 mmol kg^−1^, respectively.

### Effect of chelate on total and DTPA-extractable soil metals

**Figure 1:**
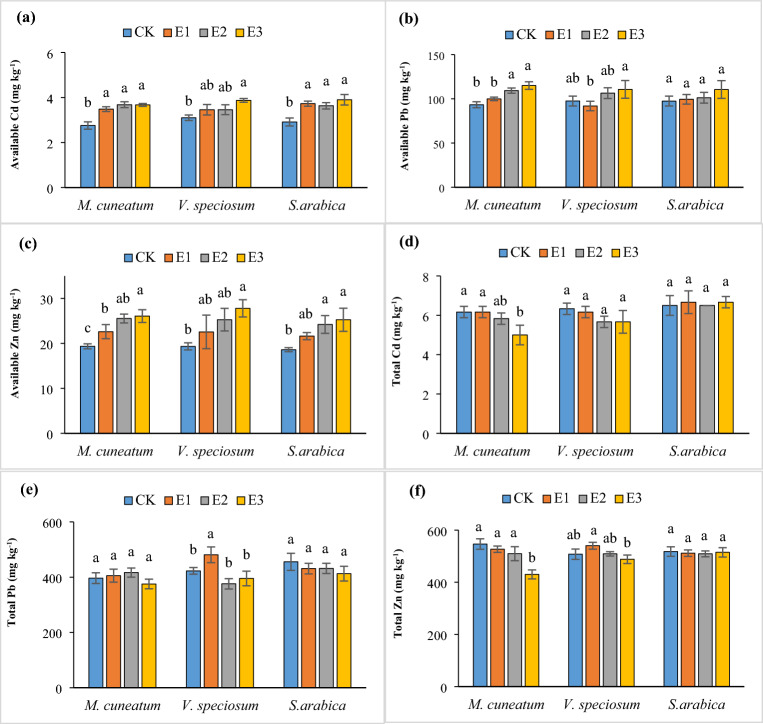
DTPA-extractable Cd (**a**), Pb (**b**), and Zn (**c**) and total content of Cd (**d**), Pb (**e**), and Zn (**f**) metals under EDTA treatment (mean ± SD; n = 3). Different letters for each attribute denote a significant difference from the corresponding control and other treatments (P < 0.05, Tukey’s test). CK, E1, E2, and E3 represent soil treated with EDTA of 0, 1, 3, and 5 mmol kg^−1^, respectively.

The total heavy metal contents of the soil after plant harvesting are presented in Fig. 1. The concentrations in control soils cultivated by *S. arabica*, *M. cuneatum*, and *V. speciosum* were Cd 6.5 mg kg^−1^, Pb 455.5 mg kg^−1^, and Zn 517.83 mg kg^−1^; Cd 6.16 mg kg^−1^, Pb 396.33 mg kg^−1^, and Zn 546.33 mg kg^−1^; and Cd 6.33 mg kg^−1^, Pb 422.83 mg kg^−1^, and Zn 507.5 mg kg^−1^, respectively (Fig. 1). Applying EDTA in most cases did not show a significant effect on the total concentration of heavy metals compared to the initial experimental soil values (Cd 6.85 mg kg^−1^, Pb 472 mg kg^−1^, and Zn 568.42 mg kg^−1^), but in general it slightly reduced their amount, especially at 3 and 5 mmol kg^−1^. In comparison with the soil without EDTA treating, EDTA significantly reduced the Cd and Zn up to 18 and 21.12%, respectively in soil planted by *M. cuneatum* (Fig. 1). In accordance with our results, Li et al.^37^ reported that the reason for the reduction of heavy metals in cultivated soil is their uptake by plants; also, these changes can be caused by EDTA's effects on soil metal species^2^. The high accumulation of heavy metals in plants and their solubility in the soil is a crucial factor in achieving phytoremediation goals^42^. Using EDTA, soil Zn, Pb, and Cd extractability significantly increased (P < 0.05) (Fig. 1). EDTA caused a promotion of 25, 19, and 34% of available Cd in the soil cultivated with *M. cuneatum*, *S. arabica*, and *V. speciosum*, respectively, compared to the untreated group (Fig. 1). However, increasing the concentration of this amendment did not show a significant effect on exchangeable Cd content; according to Liphadzi and Kirkham^43^, EDTA doses greater than 0.5 g kg^−1^ did not change the available Cd, probably due to the soil's weak adsorption of this element^44^. The highest available Pb with an increase of 23, 13, and 13% compared to the untreated soil was obtained in the soil planted with *M. cuneatum*, *S. arabica*, and *V. speciosum*, respectively, at the concentration of 5 mmol kg^−1^ EDTA (Fig. 1). Regarding Zn, its solubility in soil was directly correlated with the dose of EDTA, and the maximum value was obtained with chelating agents of 5 mmol kg^−1^ with an increase of 34, 43, and 35% compared to untreated soil for *M. cuneatum*, *S. arabica*, and *V. speciosum* respectively (Fig. 1). Due to its acidic property, EDTA reduced the adsorption of metals by soil minerals and organic clays by decreasing the soil pH and subsequently increasing the DTPA-extractable of metals^45^. Arshad et al.^46^ and Shahid et al.^47^ reported that EDTA separated Pb from mineral and organic substances in soil, increasing its availability. Also, EDTA increases their solubility in soil by forming complexes with heavy metals^2^, thereby enhancing soil-to-plant biomass metal transfer efficiency^26^.

### The uptake of Cd, Zn, and Pb by the studied species

**Figure 2:**
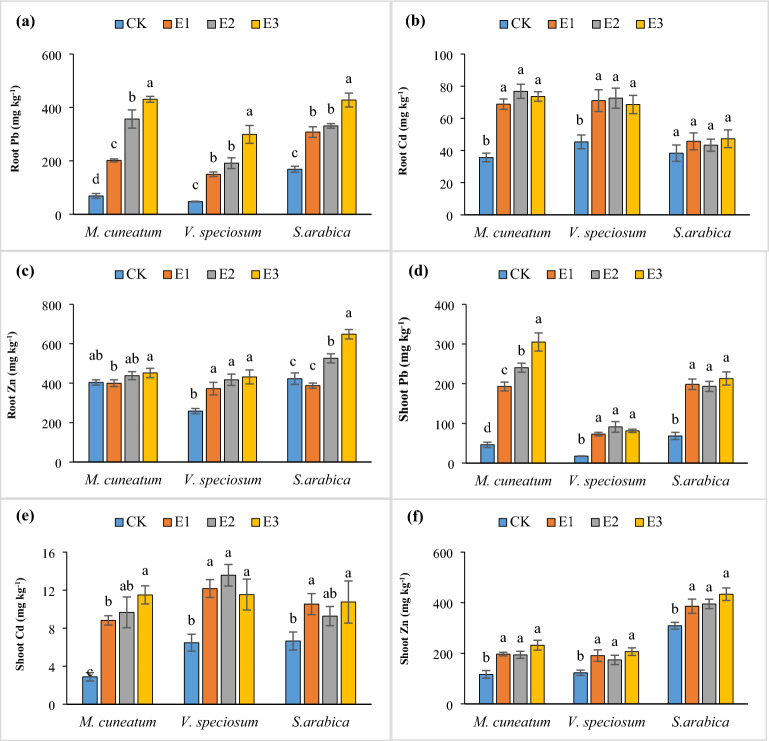
The effect of EDTA on the root Pb (**a**), Cd (**b**), and Zn (**c**) and shoot Pb (**d**), Cd (**e**), and Zn (**f**) contents of the studied species (mean ± SD; n = 3). Different letters for each attribute denote a significant difference from the corresponding control and other treatments (P < 0.05, Tukey’s test). CK, E1, E2, and E3 represent soil treated with EDTA of 0, 1, 3, and 5 mmol kg^−1^, respectively.

The concentration of Pb, Cd, and Zn in the roots and shoots of the studied species upon EDTA application is shown in Fig. 2. The root Pb content was 68.77, 47.75, and 168.1 mg kg^−1^ in the untreated group, while the aerial Pb concentration was 46.06, 17.37, and 68.26 mg kg^−1^, respectively for *M. cuneatum*, *S. arabica*, and *V. speciosum* (Fig. 2). Pb concentrations increased significantly in the roots of all three studied species when EDTA chelating agent was applied and were directly related to EDTA dose, so that at the level of 5 mmol kg^−1^, the Pb content went up by 154, 536, and 525% in *S. arabica*, *V. speciosum*, and *M. cuneatum*, respectively, compared to the control; this increase was 563, 376, and 211% in shoots (Fig. 2). The increase in the accumulation of Pb in plant tissues is due to the increased DTPA-extractable Pb content because of EDTA application^46,48^. Gul et al.^25^ claimed that the content of Pb in the roots of *Pelargonium hortorum* increased by 2.7 times in the presence of 5 mmol kg^−1^ EDTA. Also, in the study by Arshad et al.^47^ in 1000 mg kg^−1^ Pb-contaminated soil, 10 mmol kg^−1^ EDTA increased root and shoot Pb up to 7.9 and 8.6 times, respectively, in *Pelargonium hortorum*. Without EDTA treatment, the Cd content in the roots of *S. arabica*, *V. speciosum*, and *M. cuneatum* was 38.33, 45.35, and 35.66 mg Kg^−1^, respectively (Fig. 2). This concentration increased by 115, 60, and 23% with the addition of EDTA; however, no significant effect was observed between different levels of EDTA, and by increasing its dose, the absorption of Cd remained approximately unchanged. Regarding shoot Cd, EDTA enhanced its concentration from 6.65 to 10.75, 6.47 to 13.5, and 2.88 to 11.49 mg kg^−1^, with 61, 109, and 298% increases, respectively, for *S. arabica*, *V. speciosum*, and *M. cuneatum* (Fig. 2). EDTA increased the Zn content of roots from 423.01 to 648.27, 258.34 to 431.24, and 403.59 to 451.66 mg kg^−1^ by 53, 67, and 12%, respectively, for *S. arabica*, *V. speciosum*, and *M. cuneatum* compared to the untreated group; this enhancement was 40, 67, and 99% in the studied plant's shoots, respectively (Fig. 2). Using the EDTA chelating agent and as a result of the formation of metal-EDTA complexes, all the routes of entry of metals into the root, such as mass flow, absorption on the root, and binding to functional groups on the root surface, are affected^32,47,49^. As a result of the physical damage induced by the chelating agent and metal, the Pb-EDTA complex could penetrate the root endoderm as well as the Casparin strip^50^, increasing metal absorption through the roots. Investigating the xylem sap of *Hordeum vulgare* cultivated in polluted soils treated with EDTA revealed metal-EDTA complexes but not individual EDTA^51^.

### Antioxidant enzyme system activity and contents of MDA and chlorophyll

**Figure 3:**
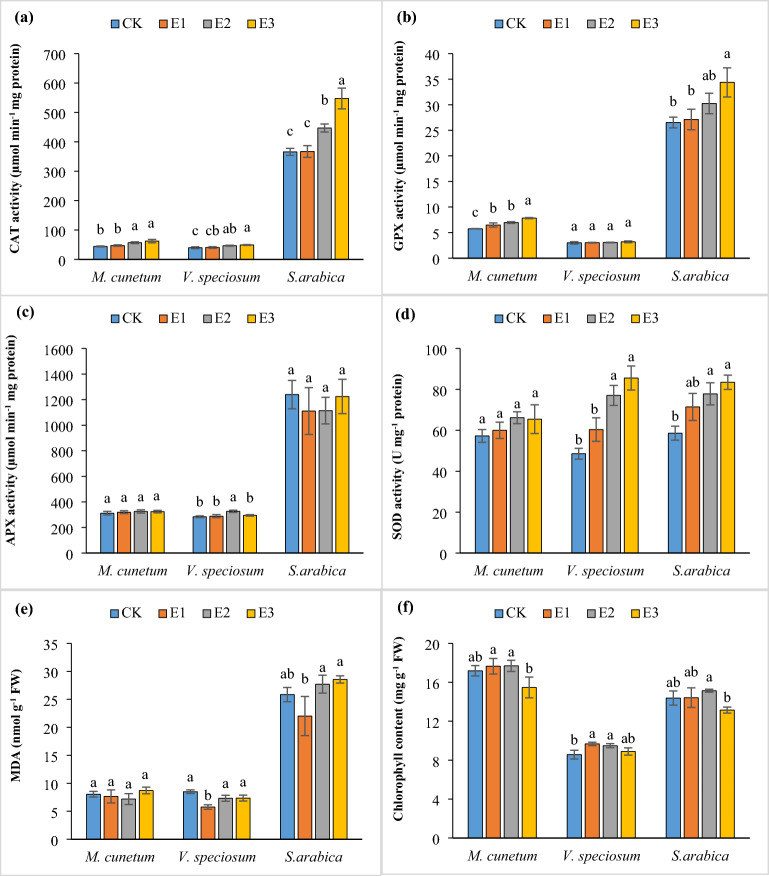
The effect of EDTA on CAT (**a**), GPX (**b**), APX (**c**), and SOD (**d**) enzyme activities and the concentration of lipid peroxidation (**e**) and total chlorophyll (**f**) of the studied species leaf (mean ± SD; n = 3). Different letters for each attribute denote a significant difference from the corresponding control and other treatments (P < 0.05, Tukey’s test). CK, E1, E2, and E3 represent soil treated with EDTA of 0, 1, 3, and 5 mmol kg^−1^, respectively.

As shown in Fig. 3, the application of an EDTA chelating agent remarkably influences the enzyme activity in the studied species. The linear increase in catalase activity was observed by adding EDTA, so that at 5 mmol kg^−1^, its activity increased by 49, 23, and 40% in *S. arabica*, *V. speciosum*, and *M. cuneatum*, respectively, compared with those grown in the untreated group (Fig. 3). Similar to catalase, the highest activity of GPX was achieved at 5 mmol kg^−1^ with 23, 7, and 26% stimulation for *S. arabica*, *V. speciosum*, and *M. cuneatum*, respectively, compared to the untreated group (Fig. 3). EDTA does not appear to have a significant effect on APX activity, and its changes did not show a clear trend when different concentrations of this chelating agent were applied. SOD activity was not significantly different between EDTA doses in *M. cuneatum*, whilst, it showed a considerable increase up to 77 and 43% for *V. speciosum* and *S. arabica*, respectively, when chelated (Fig. 3). Heavy metal concentrations increase in plants when EDTA is added, producing reactive oxygen species (ROS) like H_2_O_2_ and OH^−^. To combat ROS, SOD converts superoxide (^•^O_2_^−^) to H_2_O_2_ as a first line of defense and is decomposed by CAT into H_2_O and O_2_ to deal with oxidative stress^52,53^. Peroxidase enzymes (APX and GPX) act as a defense molecule to eliminate H_2_O_2_ and prevent peroxidative damage by reducing and catalyzing ROS during severe stress^54^. Increasing antioxidant system activity is considered to be an adaptive response to environmental stress conditions, which these enzymes create synergistically to protect plants against oxidative stress^39^. There have been several studies reporting an increase in enzymatic activity under EDTA application^11,41,55^. As a marker of lipid peroxidation, MDA is generally increased under metal stress due to increased ROS production^42,56^. In this research, EDTA demonstrated a negligible effect on the content of MDA, but in general, MDA decreased somewhat at lower concentrations (1 and 3 mmol kg^−1^). Although EDTA increased the heavy metal stress in the studied species, the increase in antioxidant enzyme activity resulted in ROS being removed. Leaf chlorophyll concentration is usually considered an effective characteristic to quantify plant physiological reactions^57^. The addition of EDTA did not show an obvious trend in the chlorophyll content of the studied species, however, its maximum amount was obtained in species *V. speciosum* and *S. arabica* at a level of 3 mmol kg^−1^, and the lowest was related to the 5 mmol kg^−1^ (Fig. 3). Saleem et al.^11^ found that applying 3 mM EDTA enhanced the chlorophyll content of *Corchorus capsularis* L. under pollution stress conditions. Also, Rathika et al.^41^ observed that EDTA significantly elevated the chlorophyll concentration in *Brassica juncea* planted in soil contaminated with Pb, while in a study, the application of 2.5 mM EDTA decreased the a, b, and total chlorophyll concentration by increasing the content of heavy metals in the organs of the species *Petunia hybrida* L.^55^ EDTA may cause oxidative stress, leading to a decrement in photosynthetic pigments^58^. Generally, in comparison with plants grown under control conditions, plants treated with EDTA showed higher levels of Cd, Pb, and Zn and produced antioxidant molecules that alleviated heavy metal-induced oxidative damage. Consequently, the studied plants have the ability to cope with Cd, Zn, and Pb stress and can be a suitable option for phytoremediation, although at 5 mmol EDTA, phytotoxicity occurred with a decrease in biomass, which should be considered.

### Phytoremediation efficiency of Pb, Zn, and Cd by studied plants

Bioconcentration, accumulation, and translocation factors of the studied species are listed in Table 2. Generally, EDTA enhanced the TF values of Pb, Zn, and Cd in all three species compared to the control, although the extent varied for the different metals and plants. The highest Cd TF values for *S. arabica*, *V. speciosum*, and *M. cuneatum* were 0.23, 0.18, and 0.15, respectively (Table 2). Regarding Pb, at a low EDTA dosage, the migration of metal to the shoot of the studied species was strengthened, but with increasing its concentration, the value of TF decreased. The highest value of TF for all studied species was found at 1 mmol kg^−1^ EDTA, so that the TF increased from 0.40 to 0.64, 0.36 to 0.48, and 0.68 to 0.95, respectively, in *S. arabica*, *V. speciosum*, and *M. cuneatum*. In comparison with the control, the chelating agent increased the TF of Zn in the studied species and induced the maximum value for *M. cuneatum* at the level of 5 mmol kg^−1^ with an increase in TF from 0.28 to 0.51, while the maximum TF between EDTA treatments for *V. speciosum* (0.51) and *S. arabica* (0.99) was observed at the concentration of 1 mmol kg^−1^.

**Table 2:** The effect of EDTA on the change of bioconcentration, translocation and accumulation factors (mean; n = 3).

Species		TF				BCF				AF			
		CK	E1	E2	E3	CK	E1	E2	E3	CK	E1	E2	E3
*M. cuneatum*	Cd	0.08^a^ ± 0.006	0.12^a^ ± 0.002	0.12^a^ ± 0.02	0.15^a^ ± 0.01	5.94^c^ ± 0.44	11.19^b^ ± 1.01	13.17^b^ ± 0.69	14.77^a^ ± 0.91	0.48^c^ ± 0.07	1.43^b^ ± 0.14	1.66^b^ ± 0.36	2.32^a^ ± 0.40
	Pb	0.68^b^ ± 0.16	0.95^a^ ± 0.03	0.67^b^ ± 0.06	0.70^b^ ± 0.05	0.17^d^ ± 0.01	0.49^c^ ± 0.02	0.86^b^ ± 0.10	1.16^a^ ± 0.16	0.11^c^ ± 0.02	0.47^b^ ± 0.03	0.58^b^ ± 0.08	0.81^a^ ± 0.10
	Zn	0.28^b^ ± 0.04	0.49^a^ ± 0.03	0.44^a^ ± 0.05	0.51^a^ ± 0.04	0.73^b^ ± 0.02	0.75^b^ ± 0.04	0.85^ab^ ± 0.04	1.06^a^ ± 0.17	0.21^c^ ± 0.02	0.37^b^ ± 0.005	0.38^b^ ± 0.04	0.54^a^ ± 0.03
*V. speciosum*	Cd	0.14^a^ ± 0.03	0.17^a^ ± 0.02	0.18^a^ ± 0.03	0.16^a^ ± 0.02	7.17^b^ ± 0.81	11.55^a^ ± 1.53	12.80^a^ ± 0.85	12.26^a^ ± 2.36	1.02^b^ ± 0.12	1.97^a^ ± 0.22	2.40^a^ ± 0.30	2.04^a^ ± 0.27
	Pb	0.36^b^ ± 0.01	0.48^a^ ± 0.006	0.47^a^ ± 0.02	0.27^c^ ± 0.01	0.11^d^ ± 0.006	0.31^c^ ± 0.05	0.51^b^ ± 0.07	0.76^a^ ± 0.11	0.04^c^ ± 0.002	0.15^b^ ± 0.02	0.24^b^ ± 0.04	0.20^ab^ ± 0.02
	Zn	0.47^a^ ± 0.04	0.51^a^ ± 0.04	0.41^a^ ± 0.05	0.47^a^ ± 0.03	0.51^b^ ± 0.04	0.69^b^ ± 0.10	0.81^a^ ± 0.04	0.89^a^ ± 0.15	0.24^b^ ± 0.02	0.35^a^ ± 0.04	0.34^ab^ ± 0.03	0.42^a^ ± 0.05
*S. arabica*	Cd	0.17^a^ ± 0.04	0.23^a^ ± 0.02	0.21^a^ ± 0.04	0.23^a^ ± 0.07	5.94^a^ ± 1.04	6.90^a^ ± 1.12	6.66^a^ ± 0.57	7.10^a^ ± 0.89	1.02^b^ ± 0.13	1.58^ab^ ± 0.16	1.42^ab^ ± 0.15	1.61^a^ ± 0.36
	Pb	0.40^c^ ± 0.07	0.64^a^ ± 0.005	0.58^ab^ ± 0.03	0.50^bc^ ± 0.06	0.37^c^ ± 0.02	0.71^b^ ± 0.06	0.77^b^ ± 0.09	1.04^a^ ± 0.10	0.15b ± 0.03	0.46a ± 0.04	0.45a ± 0.07	0.51^a^ ± 0.02
	Zn	0.73^b^ ± 0.07	0.99^a^ ± 0.09	0.75^b^ ± 0.06	0.66^b^ ± 0.03	0.81^c^ ± 0.01	0.75^c^ ± 0.04	1.03^b^ ± 0.06	1.26^a^ ± 0.08	0.59^b^ ± 0.05	0.75^a^ ± 0.05	0.77^a^ ± 0.04	0.84^a^ ± 0.03

Different lowercase letters in a same row indicate significant differences existed among different plants or soils (P < 0.05, Tukey’s test). CK, E1, E2, and E3 represent soil treated with EDTA of 0, 1, 3, and 5 mmol kg^−1^, respectively.

The distribution percentage of metals revealed that the metal accumulation in the above-ground parts of the studied species is less than 50% (Table 3). Generally, EDTA has been found to improve the metal distribution rate in the shoots. In most cases, 1 mmol kg^−1^ EDTA was more effective for metal distribution in the shoots than other doses, which indicates that increasing the dose causes more accumulation in the roots. In fact, as the concentration of EDTA increases, the metal content in the roots increases, and most likely, the plant's roots prevent metals from emigrating into the aerial organs. With respect to this, Wei et al.^72^ pointed out that the roots of *Sorghum sudanense* reduced the transfer ability of some metals to the shoots by binding metals to the root walls. Metal translocation into the plant's shoot may be limited due to obstruction of the casparian strip, accumulation in plasma membranes, deposition in intercellular spaces, deposition of insoluble salts, or fixation in root vacuoles and nodule cells^47^. The degradation of the root membrane by EDTA probably increases the mobility of metals into the plant's aerial parts^50^. In this regard, Kamal et al.^35^ stated that using EDTA decreased the metal accumulation in the root and transferred it more to the shoot. Moreover, EDTA increases the infusion of root metals into the vascular tissue via the apoplast, thereby enhancing their uptake by shoot tissues^59^. However, the increase in translocation by EDTA for the studied plant did not follow a clear trend, reflecting the complex nature of the metal transfer process in plants^38^. *M. cuneatum* showed a higher shoot distribution for Pb than other species, while *S. arabica* was more effective in the translocation of Cd and Zn. In our previous study, this plant, with a TF of 4.1 for Pb, showed the best phytoextraction capability among the 25 native species^9^. Also, other researchers have observed significant levels of heavy metals, including Zn, Pb, Cd, chromium (Cr), mercury (Hg), and tungsten (W), in tissues of the genus *Marrubium*, demonstrating their suitability as candidates for remediation goals^59–64^. The high phytostabilization potential of *Stipa. barbata*, another species of the genus *Stipa*, with BCF = 2.5 and 1.92, respectively, for Cd and Zn in cleaning soils contaminated with these metals, has been mentioned^65^. Consistent with our results, in a study investigating the lead phytoremediation ability of five bamboo species in the presence of EDTA, Jiang et al.^2^ found differences in metal accumulation, and *Arundinaria argenteostriata* and *Arundinaria fortunei* had higher Pb migration to shoots compared to other species. Actually, this indicates the different potential of plants to uptake metals in their tissues, emphasizing the need to examine the accumulation behavior of plants with and without chelating agents to determine how they compartmentalize different metals in their tissues.

**Table 3:** Effects of the EDTA application on the shoot distribution rate of metals in the studied species (mean; n = 3).

Species	Metal distribution (%)								
	*M. cuneatum*			*V. speciosum*			*S. arabica*		
	Pb	Zn	Cd	Pb	Zn	Cd	Pb	Zn	Cd
CK	40 ± 5.56	22.33 ± 3.05	7.66 ± 0.57	26.66 ± 1.15	32 ± 1.73	12.66 ± 2.88	28.66 ± 3.51	42.33 ± 2.51	15.33 ± 3.51
E1	49 ± 1.00	33.33 ± 1.52	11.33 ± 0.57	32.66 ± 0.57	34 ± 2.00	14.66 ± 1.52	39 ± 0.00	49.66 ± 2.08	18.66 ± 1.52
E2	40.33 ± 2.08	30.33 ± 2.51	11 ± 2.00	32 ± 1.00	29.33 ± 2.30	15.66 ± 2.08	36.66 ± 1.52	42.66 ± 2.08	18 ± 2.64
E3	41.33 ± 1.52	34 ± 2.64	13.66 ± 1.52	21.33 ± 1.52	32.33 ± 1.52	14.67 ± 2.08	33 ± 2.64	40 ± 1.00	18.66 ± 5.03

CK, E1, E2, and E3 represent soil treated with EDTA of 0, 1, 3, and 5 mmol kg^−1^, respectively.

EDTA's effect on BCF of Pb, Cd, and Zn was incremental, and there was a linear relationship with the dosage. The value of BCF for Cd was considerably high in all three studied species and reached its maximum value at 5 mmol kg^−1^ EDTA with 7.10, 12.26, and 14.77 for *S. arabica*, *V. speciosum*, and *M. cuneatum*, respectively. This amendment increased the BCF of Pb in *M. cuneatum* and *S. arabica* to above 1 (BCF > 1), and with a tremendous increase from 0.11 to 0.76, it remained below one for *V. speciosum*. The BCF value of Zn by adding 5 mmol kg^−1^ EDTA for *M. cuneatum* and *S. arabica* reached 1.06 and 1.03 (BCF > 1), respectively, showing their stabilization potential for Pb and Zn-contaminated soils. Species with BCF > 1 and TF < 1 are most suitable for phytoremediation purposes through phytostabilization^66^. The hyperaccumulator *Pelargonium hortorum* with BCF > 1 was introduced for phytostabilization of lead-contaminated soils after EDTA treatments^46^. Regardless of the application of EDTA or without it, all species studied reflected BCF > 1 and TF < 1 for Cd, demonstrating a good capability for cadmium remediation via phytostabilization^67^. *M. cuneatum* with an BCF > 1 and a TF < 1 has been recommended as a species to clean up Cd-contaminated soil through the phytostabilization strategy^9^. Also, Tananonchai et al.^66^ reported that *Pennisetum purpureum*, a Cd hyperaccumulator with TF < 1, can be regarded as a Cd stabilizer, when combined with chelating agents. Similar findings of BCF with 0.55^68^, 0.42^69^, and 0.47^9^ were reported for Zn in *V. speciosum*; these values for TF were 0.12, 2.32, and 1.5, respectively. Hosseinniaee et al.^9^ reported the BCF and TF of 0.42 and 0.98, and 0.23 and 2.32, respectively, for Pb and Cd in *V. speciosum*. In the study of Malayeri et al.^65^ *V. speciosum* had a TF of 5.91, 1.5, and 1.16 for Zn, Pb, and Cd, respectively, while BCF < 1 for all three metals. Although there is limited literature pertaining to *S. arabica* phytoremediation properties, it was recently found to be a suitable option for remediation of contaminated sites with TF > 1 for Pb, Zn, and Cr^9^. Some discrepancies in the phytoremediation factors of the studied plants with the literature are due to the different growth stages, growth conditions, and concentrations of heavy metals in the soil of their habitat.

EDTA application enhanced the AF of studied species, and except in a few cases, this increase was consistent with the dosage. The AF value of Cd in *M. cuneatum* at all levels of the chelating agent and even for *V. speciosum* in the control treatment was higher than one (AF > 1). This amendment increased the AF of Pb from 0.15 to 0.51, 0.04 to 0.24, and 0.11 to 0.81 for *S. arabica*, *V. speciosum*, and *M. cuneatum*, respectively. EDTA also raised the AF value of Zn for the studied species, although the effect was not as strong as that of Pb and Cd. The maximum AF values of Zn were 0.84, 0.42, and 0.54 in *S. arabica*, *V. speciosum*, and *M. cuneatum*, respectively, at 5 mmol kg^−1^ EDTA. As opposed to TF, the value of BCF as well as almost AF of the studied plants improved with increasing EDTA concentration; these results are compatible with the findings of Guo et al.^7^ and Li et al.^37^. Plants with AF and TF > 1 and AF and TF < 1 display accumulator and excluder properties, respectively^70,71^. According to this, the studied plants are considered excluders of Pb, Zn, and Cd and cannot be hypeaccumulators based on Reeves et al.^72^ criteria; however, with respect to Cd, they demonstrated AF > 1, but still showed TF < 1. Although researchers claimed that, except for hyperaccumulators or chelate-assisted plants, approximately 95% of the metals, especially Pb, adsorbed by plants will be retained in the roots^73^. From this perspective, the distribution of Pb in shoots of *M. cuneatum* was higher than 0.4, showing the strong capability of this species in phytoremediation of Pb-contaminated lands alone or in combination with EDTA. In contrast, *S. arabica* and *V. speciosum* were more suitable for removing Zn and Cd from polluted soil, respectively via the phytoextraction process. On the other hand, the partitioning of metals in plants is regulated by different metabolic processes and depends on a plant's growth stage, probably making it impossible to determine definitively whether the plant is an accumulator or excluder based on laboratory and greenhouse data. Under long-term cultivation conditions, a large amount of metal accumulates in plants^10^. Furthermore, there is much scientific evidence showing that when hyper-accumulators are grown in pot experiments by acquiring AF < 1, they exhibit excluder behavior^7,11,46^. As a result, the accumulation of metals in plants' tissues in both laboratory and field conditions, with different planting periods, needs to be considered in the further studies. Generally, EDTA effectively increased the phytoextraction ability of studied species for the investigated metals, but this enhancement was not the same for all metals and species. It is unlikely that *V. speciosum* would be suitable for phytoextraction of lead because of the low Pb concentration in the shoot, despite the EDTA application, while displaying a notable capacity for Cd absorption and accumulation, making it an effective Cd remediator when compared to other species. *M. cuneatum*, which has the potential to produce high biomass under a variety of soil types and climates with its woody root system and numerous stems, exhibited a significantly stronger response in the translocation of Pb to the shoot in the presence of EDTA. Thus, it could be an appropriate candidate for the Pb phytoextraction strategy. *S. arabica* without applying EDTA revealed the best performance in adsorbing and transferring all three metals Cd, Pb, and Zn, and this potential was strengthened along with EDTA. In addition to its long-term association with human activity, *S. arabica* is widely distributed, requires low ecological attention, is resistant to grazing and environmental conditions, and is considered a valuable soil stabilizer and arid land ecology model system^74^. Therefore, this plant can act as a phytoremediator in restoring multi-metal-contaminated soils, especially in arid and semi-arid regions. Briefly, it can be concluded that *S. arabica* and *V. speciosum* species presented the highest and lowest Zn absorption and translocation capacities, respectively. Regarding Pb, the accumulation and translocation ability of *M. cuneatum* for this element was higher than other studied species, and the lowest was related to *V. speciosum*. The species of *V. speciosum* was more effective in accumulating Cd in its organs than other species, followed by *M. cuneatum*.

### Optimization of EDTA concentration

**Figure 4:**
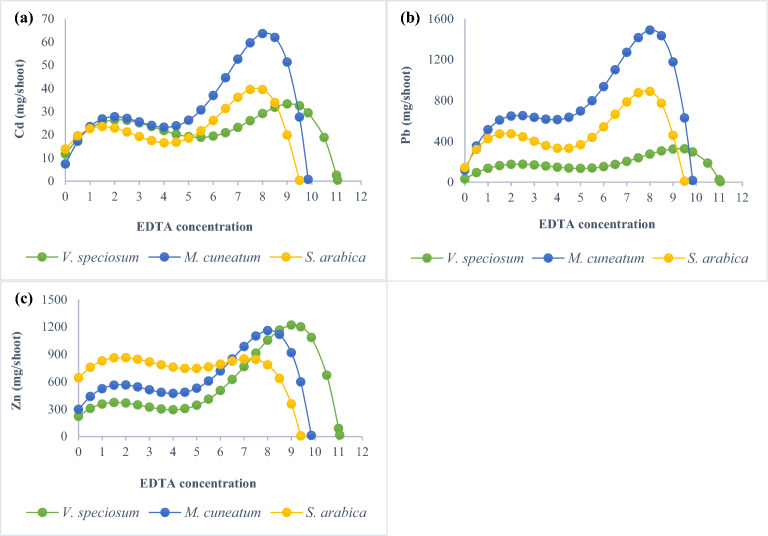
Optimum dose of EDTA application for Cd (**a**), Pb (**b**), and Zn (**c**) absorption based on shoot biomass and its metal concentration.

The optimum EDTA value in the absorption process for the studied plants and heavy metals is shown in Fig. 4. The results revealed that the values of 9.1, 8.15, and 7.8 mmol kg^−1^ of EDTA are the suitable concentrations for Cd extraction by *V. speciosum*, *M. cuneatum*, and *S. arabica*, respectively. Regarding Pb, the highest EDTA levels which induced the optimal response were 9.2, 8.1, and 7.8 mmol kg^−1^ for *V. speciosum*, *M. cuneatum*, and *S. arabica*, respectively. For Zn extraction, EDTA concentrations of 9.1, 8.1, and 1.8 mmol kg^−1^ were the most effective by *V. speciosum*, *M. cuneatum*, and *S. arabica*, respectively. With a further increase in the EDTA dose, the decreasing growth of the plant overcomes the increasing metal concentration in the shoots, until eventually the plant dies. In fact, considering the toxicity of EDTA for plants, especially at high concentrations, it is the plant's growth characteristics that control the final amount of metal absorption. Therefore, to determine the optimal concentration of EDTA for phytoextraction purposes, a balance must be maintained between plant growth and heavy metal accumulation^50^. The results indicate that higher EDTA concentrations do not increase the amount of Zn extracted by *S. arabica*. Also, the increasing trend of extracted Pb by *V. speciosum* under EDTA showed a mild rate, while the application of EDTA caused a significant increase in the amount of metals adsorbed by *M. cuneatum*. As a result, the EDTA chelating agent's effect differs from plant to plant and even from metal to metal adsorbed by a given plant, making a case-by-case analysis necessary. Overall, EDTA effectively enhanced the extraction potential of all three metals; in most cases, the optimized concentration was higher than the doses tested in this study, and its effect was as follows: Pb > Cd > Zn for metals, and for the species, the order was *M. cuneatum* > *V. speciosum* > *S. arabica*. Nevertheless, due to their low biodegradability, EDTA and its complexes can be toxic to plants and soil microorganisms. To address these concerns and promote environmental safety, future research is needed to gain a comprehensive understanding of the processes involved in metal-EDTA complex formation and their interactions with soil physiochemical properties in order to design more effective phytoremediation strategies that minimize potential negative impacts on the environment. On the other hand, for the successful implementation of phytoextraction, it is necessary to take a comprehensive approach that includes shoot removal, proper waste management strategies, monitoring regrowth, and assessing long-term sustainability and cost-effectiveness.

## Conclusion

Plant properties and DTPA-extractable metal in soil greatly influence phytoextraction. This study found that EDTA increased the availability of heavy metals in the soil, which subsequently enhanced their absorption by the studied plants. Generally, the metals uptake by plants increased with the increase in the dose of EDTA, but at 5 mmol kg^−1^ EDTA phytotoxicity was evident due to the reduction in biomass production. To deal with oxidative stress, CAT and SOD increased significantly, GPX, although generally increased, but showed no particular change, especially in *M. cuneatum* and *V. speciosum*, while APX decreased in *S. arabica* and *M. cuneatum* and increased smoothly in *V. speciosum*. EDTA significantly increased BCF, AF and TF factors in the studied species, reflecting the high potential of this chelate for absorption purposes. In this study, *S. arabica*, *M. cuneatum* and *V. speciosum* showed the highest capability to accumulate and translocate Zn, Pb and Cd, respectively. Based on the prediction model, approximately the values of 9, 8 and 7 mmol kg^−1^ soil of EDTA can be recommended as the optimum concentration of EDTA for *V. speciosum*, *M. cuneatum*, and *S. arabica*, respectively, which result the highest extraction. Therefore, due to its stable and highly soluble metal-EDTA complex, EDTA serves as an effective chelating agent to increase metal dissolution, absorption, and translocation; also, strengthening the phytoremediation potential of the species studied in this research. However, to validate the findings of the present study, further studies should be conducted under various ecological field conditions and given the potential environmental impacts, it is important to use EDTA judiciously and consider alternative chelating agents or methods when possible. Also, regarding the medicinal and fodder nature of the studied plants and their good potential for metal stabilization, using organic amendments such as biochar and compost can be considered to investigate their effect on reducing the risk of heavy metals being transferred via food chains and develop a perspective on phytostabilization.

## Material and methods

### Growth conditions and treatments

The soil polluted with Zn, Pb, and Cd was collected from the Angouran Pb–Zn mine site, Iran (36°36′41″ N and 47°23′32″ E), air dried, sieved (≤ 2 mm) for removing gravel, stone, and plant debris, and homogenized. The physico-chemical characteristics of the experimental soil are presented in Table 4. The loamy soil pH was slightly alkaline (pH 7.12) with an electrical conductivity (EC) of 0.31 ds m^−1^. The used soil, with organic matter (OM%) of 2.33% and content of 0.19%, 41.6, and 494 mg kg^−1^, respectively, for total nitrogen (N%), available phosphorous (P), and soluble potassium (K), was fertile in terms of crop cultivation. Total Zn, Pb, and Cd concentrations were 568.42, 472, and 6.85 mg kg^−1^, respectively, which far exceeded their background values (20, 95, and 0.3 for Pb, Zn, and Cd, respectively)^75^ and the maximum allowed concentration limits (100, 300, and 3 for Pb, Zn, and Cd, respectively)^76^, indicating that the experimental soil is severely contaminated and needs to be remediated. The seeds of *S. arabica*, *M. cuneatum*, and *V. speciosum* species were purchased from the Research Institute of Forests and Rangelands, Tehran. After being surface sterilized with 5% sodium hypochlorite for 10 min, the seeds were washed several times with distilled water. Due to the herbaceous life form of species *M. cuneatum* and *V. speciosum*, one seedling per pot was sawn. Regarding *S. arabica*, because of the bunch growth system of this species, to produce appropriate biomass, 0.1 g were planted. Each plastic pot was 18 × 17 cm, containing approximately 3000 g of contaminated soil. A greenhouse experiment was carried out from 22 August to 22 February for six months on the Agriculture and Natural Resources Campus of Tehran University in a growth chamber with day (25 ± 5 °C) and night (15 ± 5 °C) temperatures and soil humidity of 60–80% of the field water-holding capacity. Three replications of each treatment were conducted in a completely randomized design, and 36 pots were divided into nine experimental groups randomly. A plant's growth and development are influenced by chelating agents, and EDTA has not been examined for its effect on the studied plants. Hence, dosages of 0 (CK), 1 (E1), 3 (E2), and 5 (E3) mmol EDTA kg^−1^ soil were selected according to previous studies^37,46^, and 50 days before harvesting, along with irrigated water, were applied in all experiments.

**Table 4:** Physical–chemical properties of experimental soil.

Characteristic	Value
pH	7.12
EC (ds m^−1^)	0.31
Total N (%)	0.19
OM (%)	2.33
Texture	Loamy
Phosphorous (P)	
Total (%)	0.06
Soluble (mg kg^−1^)	41.6
Potassium (K)	
Total (%)	0.44
Soluble (mg kg^−1^)	494
Total Zn (mg kg^−1^)	568.42
Total Pb (mg kg^−1^)	472
Total Cd (mg kg^−1^)	6.85

### Plant harvesting and sampling

One day before harvest, the required amount of leaves from each plant were sampled in the pot and instantly transferred to liquid nitrogen, which was then kept at − 70 °C for physiological analysis such as antioxidant enzyme activities, chlorophyll, and malondialdehyde. The plants were collected from the pots after 6 months of sowing and split into shoots and roots. To remove the adhering soil particles, they were repeatedly washed with tap water and finally rinsed using de-ionized water. The dry matter of the roots and shoots was recorded after drying at 70 °C in the oven for 48 h (to reach a constant weight). Soil samples were air-dried and sieved with a 2 mm mesh for chemical analysis.

### Measuring soil properties and determining the concentration of heavy metals in soil and plants

Rhizosphere pH and EC were measured in a suspension of soil and water (1:2.5 ratio), available K and extractable P using a flame photometer and spectrophotometer (Shimadzu UV-160), respectively^77^, total nitrogen by the Kjeldahl method^78^ and a Walkley–Black titration was used to determine the OM^79^. Regarding the metal analysis in soil, the total concentration was extracted by digesting samples with a solution composed of HNO_3_ and HCL (3:1 ratio)^80^, and DTPA-extractable metals were determined using diethylenetriamine penta-acetic acid (DTPA) 0.005 M^81^. In order to extract heavy metals from plants, 0.2 g of dried powder was heated in a furnace at 500 °C for 6 h, then transferred into a beaker with 20 mL of 1 N HCl and kept at 150 °C for 20 min. Final filtration and dilution with distilled water to 100 mL are performed. Eventually, the contents of heavy metals in soils and plants were measured using the ICP-OES (Spectro Arcos-Germany, 1999).

### Translocation factor (TF), accumulation factor (AF), and bioconcentration factor (BCF)

For investigating the accumulation and translocation of each metal in plants, TF (the ratio of metal content in shoots to the concentration of that in the roots) (Eq. (1))^82^, BCF (root metal content ratio to that in rhizosphere soil) (Eq. (2))^83^, and AF (the proportion of the shoot metal concentration to its content in the soil) (Eq. (3))^84^ were calculated. Higher values of BCF, AF, and TF display a stronger ability of a certain metal to migrate from the soil to the plant's root and then to the aboveground parts. Also, the distribution percentage of metal in the aerial part (shoot metal concentration/total metal content in the plant) was investigated.

1 $$\mathrm{TF}=\left[{C}_{above}/{C}_{root}\right]$$

2 $$\mathrm{BCF}=\left[{C}_{root}{/C}_{soil}\right]$$

3 $$\mathrm{AF}=[{C}_{above}/{C}_{soil}]$$

where ${C}_{soil}$ is the total soil's metal concentration and ${C}_{above}$ and ${C}_{root}$ represent the metal contents in the shoot and root, respectively.

### Analyzing chlorophyll and oxidative stress

To determine chlorophyll content, 0.1 g of a fresh leaf sample was thoroughly ground in a mortar with 10 ml of 80% acetone and kept in the dark for 24 h at a temperature of 4 °C. Then, the extract was centrifuged for 10 min at a speed of 5000 × *g*, and the adsorbance was determined at 645, 480, and 663 nm using a spectrophotometer (Shimadzu UV-160)^85^. The concentration of membrane lipid peroxidation was measured based on malondialdehyde accumulation in leaves using thiobarbituric acid. Optical absorption was determined at wavelengths of 450, 532, and 600 nm in a spectrophotometer (ShimadzuUV-160)), and the content of MDA was obtained using its extinction coefficient (155 mM^−1^ cm^−1^) in nmol g^−1^ fresh weight (FW)^86^.

### Influence of EDTA on antioxidant enzyme activities

The enzymatic extract was gathered by crushing 0.1 g of fresh tissue in liquid nitrogen and adding 1 mL of sodium phosphate buffer (50 mM, pH 7). After centrifuging the homogenate for 20 min at 10,000 rpm and 5 °C, the activity of CAT, GPX^87^, and APX^88^ was measured from the obtained supernatant; the activities were expressed in µmol min^−1^ mg protein. The superoxide dismutase (SOD) activity was measured based on^89^ at 560 nm. The main reaction buffer included 100 mM phosphate buffer (pH 7.8), 26 mM methionine, 20 μM riboflavin, 0.1 μM EDTA, 750 μM nitroblue tetrazolium (NBT), and 20 μL of enzyme extract; expression of enzyme activity in U mg^−1^ protein.

### Optimizing the EDTA concentration

In order to achieve the optimal dose of EDTA for phytoextraction purposes, regression analysis was conducted to establish the regression relationship between EDTA concentration and both aerial biomass and aerial metal concentration of each plant. The relationship between EDTA concentration and shoot heavy metals and aerial biomass was quadratic (Eq. (4)) and cubic (Eq. (5)), respectively, and statistical model fit was evaluated using the lack of fit test. Using regression relationships, predicted values were obtained separately for shoot biomass and its metal concentration. As the amount of metal extraction is determined by the plant's aerial biomass and its metal content, those two data sets were combined to obtain the final prediction model.

4 $$Y= \beta 0+{\beta }_{1} x+{\beta }_{2} {x}^{2}+\epsilon$$

5 $$Y= \beta 0+{\beta }_{1} x+{\beta }_{2} {x}^{2}+{\beta }_{3} {x}^{3} +\epsilon$$

### Statistical analysis

After performing a normality test (Kolmogorov–Smirnov) on the data for comparing soil and plant parameters based on significance differences (P < 0.05), a one-way analysis of variance (one-way ANOVA) to examine the effects of treatments on the various characteristics analyzed was applied. Afterwards, using the Tukey test, confidence intervals were formed for all pairwise differences between the means of each group. SAS 9.4 software was used to analyze all the statistical analysis.

### Research involving plants statement

The seeds used in this study came from commercial and rangeland plants, so they were neither exotic nor endangered, under controlled conditions according to institutional, national, and international guidelines.

### Permission to collect plants

The seeds were purchased from the gene bank of Research Institute of Forests and Rangelands in Tehran, Iran. During all experiments and studies, all rules and regulations were followed by the authors.

## Data Availability

Datasets and other materials obtained during this study are available from the corresponding author and will be accessible at any time upon request.
